# Array-based assay detects genome-wide *5-mC* and *5-hmC* in the brains of humans, non-human primates, and mice

**DOI:** 10.1186/1471-2164-15-131

**Published:** 2014-02-13

**Authors:** Pankaj Chopra, Ligia A Papale, Andrew T J White, Andrea Hatch, Ryan M Brown, Mark A Garthwaite, Patrick H Roseboom, Thaddeus G Golos, Stephen T Warren, Reid S Alisch

**Affiliations:** 1 Department of Psychiatry, University of Wisconsin–Madison, 6001 Research Park Blvd., Madison, Wisconsin 53719, USA; 2 Departments of Human Genetics, Emory University School of Medicine, 615 Michael Street, Atlanta, Georgia 30322, USA; 3 Departments of Biochemistry, Emory University School of Medicine, 615 Michael Street, Atlanta, Georgia 30322, USA; 4 Departments of Pediatrics, Emory University School of Medicine, 615 Michael Street, Atlanta, Georgia 30322, USA; 5 Departments of Comparative Biosciences, University of Wisconsin – Madison, 1223 Capitol Court, Madison, Wisconsin 53715, USA; 6 Obstetrics & Gynecology, University of Wisconsin – Madison, 1223 Capitol Court, Madison, Wisconsin 53715, USA; 7 Departments of Wisconsin National Primate Research Center, University of Wisconsin – Madison, 1223 Capitol Court, Madison, Wisconsin 53715, USA

**Keywords:** Epigenetics, DNA methylation, 5-hydroxymethylcytosine (5-hmC), Evolution

## Abstract

**Background:**

Methylation on the fifth position of cytosine (*5-mC*) is an essential epigenetic mark that is linked to both normal neurodevelopment and neurological diseases. The recent identification of another modified form of cytosine, 5-hydroxymethylcytosine (*5-hmC*), in both stem cells and post-mitotic neurons, raises new questions as to the role of this base in mediating epigenetic effects. Genomic studies of these marks using model systems are limited, particularly with array-based tools, because the standard method of detecting DNA methylation cannot distinguish between *5-mC* and *5-hmC* and most methods have been developed to only survey the human genome.

**Results:**

We show that non-human data generated using the optimization of a widely used human DNA methylation array, designed only to detect *5-mC*, reproducibly distinguishes tissue types within and between chimpanzee, rhesus, and mouse, with correlations near the human DNA level (R^2^ > 0.99). Genome-wide methylation analysis, using this approach, reveals 6,102 differentially methylated loci between rhesus placental and fetal tissues with pathways analysis significantly overrepresented for developmental processes. Restricting the analysis to oncogenes and tumor suppressor genes finds 76 differentially methylated loci, suggesting that rhesus placental tissue carries a cancer epigenetic signature. Similarly, adapting the assay to detect *5-hmC* finds highly reproducible *5-hmC* levels within human, rhesus, and mouse brain tissue that is species-specific with a hierarchical abundance among the three species (human > rhesus >> mouse). Annotation of *5-hmC* with respect to gene structure reveals a significant prevalence in the 3'UTR and an association with chromatin-related ontological terms, suggesting an epigenetic feedback loop mechanism for *5-hmC*.

**Conclusions:**

Together, these data show that this array-based methylation assay is generalizable to all mammals for the detection of both *5-mC* and *5-hmC*, greatly improving the utility of mammalian model systems to study the role of epigenetics in human health, disease, and evolution.

## Background

DNA methylation and histone modifications are essential epigenetic components in the establishment of the transcriptional state of eukaryotic genes throughout the genome. The best understood of these epigenetic modifications is DNA methylation, which occurs primarily at cytosines located 5' to guanosine in the CpG dinucleotide of differentiated eukaryotic nuclei [1]. This modification is less common in CpG-rich areas, known as CpG islands, which are often located in the promoter regions of many genes and involved in transcriptional regulation. While DNA methylation is a hallmark of human cancer [2], the identification of a growing number of human diseases resulting from epigenetic disruptions emphasizes the importance of this mark [3-7].

The recent identification of another modified form of cytosine, 5-hydroxymethylcytosine (*5-hmC*), in both stem cells and post-mitotic neurons, raises new questions as to the role of this base in mediating epigenetic effects. *5-hmC* is mediated by members of the ten-eleven translocation (TET) family of proteins, which each contain a C-terminal oxidase domain. Recently, a highly efficient and selective chemical approach to label and capture *5-hmC* revealed the first distribution map of *5-hmC* in a mammalian brain genome (mouse) and showed that *5-hmC* is enriched in the gene bodies of active or highly transcribed genes [8]. Furthermore, acquisition of *5-hmC* in specific gene bodies occurs during neuronal differentiation and maturation, indicating that this mark may be distributed in a spatial and temporal manner in brain during development.

Traditionally, non-human studies of DNA methylation have relied heavily on the ‘gold standard’ for DNA methylation detection: treatment of genomic DNA with sodium bisulfite, amplification of the gene(s)/region(s) of interest, and sequencing the resulting amplicons [9]. While this method is robust, it is subject to amplification biases and considered very laborious, allowing only for a limited number of sites to be interrogated at a time. The introduction of genome-wide arrays and ‘Next-Generation’ sequencing technologies permits genome-wide interrogation of 5-methylcytosine (*5-mC*) but these methodologies either focus solely on human studies (arrays), do not provide base resolution (enrichment techniques), or are cost prohibitive (whole genome sequencing). In addition, these methods could not distinguish between *5-mC* and *5-hmC*; however, numerous sequencing-based methods have recently emerged that now allow for the quantitative discrimination of the two modified bases [10,11]. In this study we combined these recent *5-hmC* detection methods with standard array-based methods to develop a novel use for the HumanMethylation450 BeadChip from Illumina to measure both *5-mC* and *5-hmC* in mammalian model systems.

Non-human primates (e.g. rhesus monkeys) and humans have structural and functional similarities in the neural circuits mediating neuropsychiatric disease. Access to relevant brain regions associated with neuropsychiatric disorders from a monkey model improves our ability to translate research findings to humans and provides opportunities to test hypotheses that cannot be tested in humans. However, genome-wide molecular approaches, such as array based assays, in non-human genomes have not matched resources aimed at the human genome. Here, we optimized an established human array that was originally designed to detect *5-mC* levels at nearly half a million loci throughout the human genome and show that it is also capable of measuring genome-wide DNA methylation levels in other species, including non-human primates and mice.

## Results

### Assay optimization

To overcome the paucity of molecular tools for DNA methylation detection in non-human primates, we optimized a highly reproducible and widely used human array [12-16] to measure genome-wide DNA methylation levels in rhesus macaque (*Macaca mulatta*). The human array is designed to provide a quantitative measure of 5-methylcytocine (DNA methylation; *5-mC*) at 485,512 CpG dinucleotides located proximal to nearly all human RefSeq genes (N = 21,231). We interrogated each fifty nucleotide probe sequence on the array to determine the fraction that had either an exact match or up to four mismatches to the rhesus genome and found that approximately 7% and 30%, respectively, of the array probes met this criteria, (rhesus-competent probes; exact match: N = 35,901; mismatch: N = 154,030; Additional file 1; see Methods). We hypothesized that both sets of these rhesus-competent probes should reproducibly provide a quantitative measure of DNA methylation at the majority (exact match: > 55%, N = 11,804; mismatch > 90%, N = 19,362) of the human annotated RefSeq genes in the rhesus genome. Indeed filtering the array probes to only the rhesus-competent probes reduces the median number of CpG dinucleotides interrogated per gene from fifteen (human) to two or six (rhesus exact match or mismatch, respectively; Additional file 2A).

**Figure 1:**
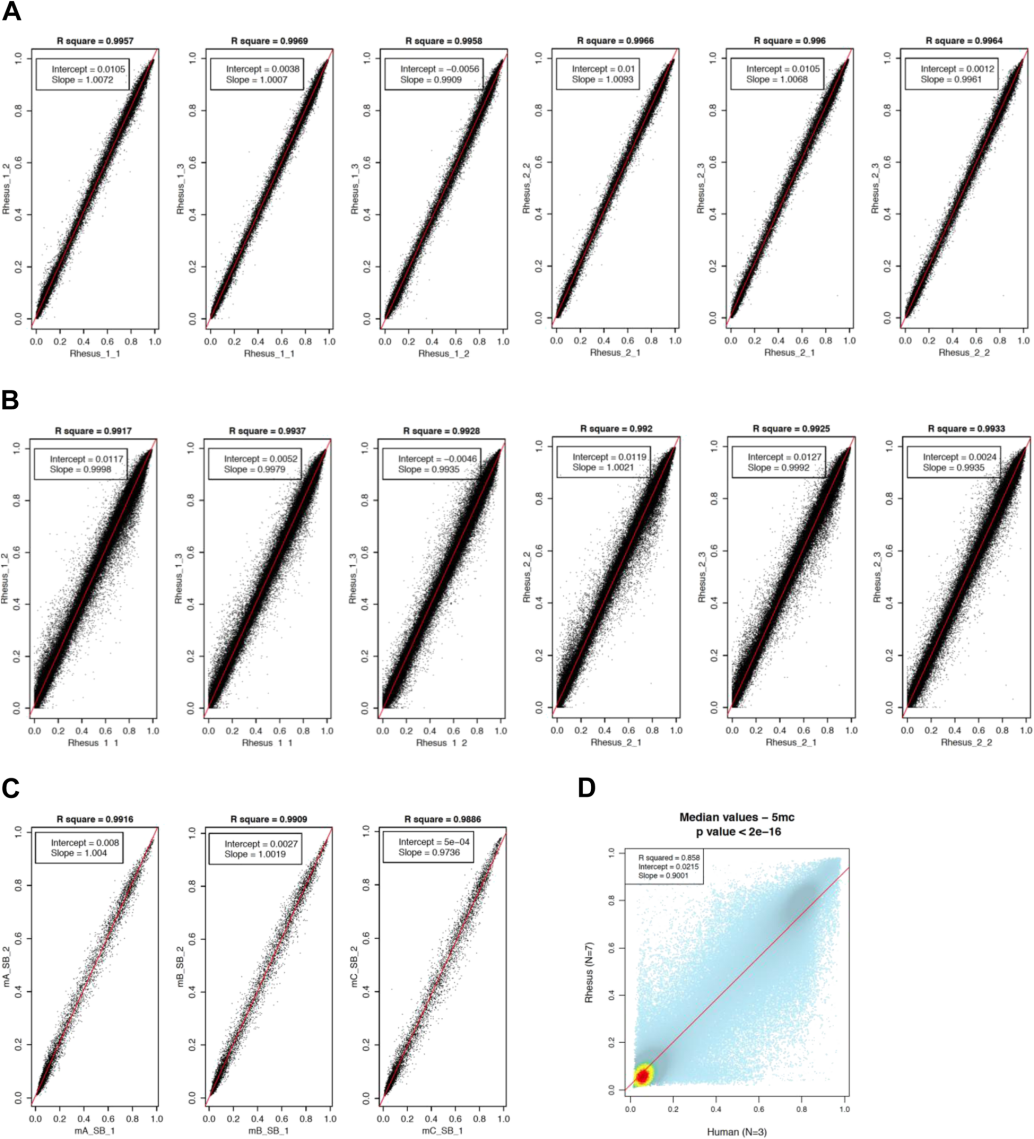
**Rhesus and mouse DNA methylation levels detected on the human array are highly correlated.** Scatter plots of rhesus **(A-B)** and mouse **(C)** methylation data (*5-mC*) generated from technical replicates run on the HumanMethylation450 BeadChips are shown for exact match (**A**; N = 35,901) and mismatch (**B**; N = 154,030) rhesus-competent probes, or mismatch mouse-competent probes (**C**; N = 9,734). **D)** Scatter plot of the *5-mC* levels of the rhesus-competent probes compared to the levels found in humans. Each probe is represented as a colored dot that are mostly light blue but become dark blue, green, yellow, or red as the density of loci increases. For all plots, the diagonal red line indicates the regression line and the x and y-axes indicate the methylation level for each replicate **(A-C)** or the median methylation values of the samples for each species **(D)**.

**Figure 2:**
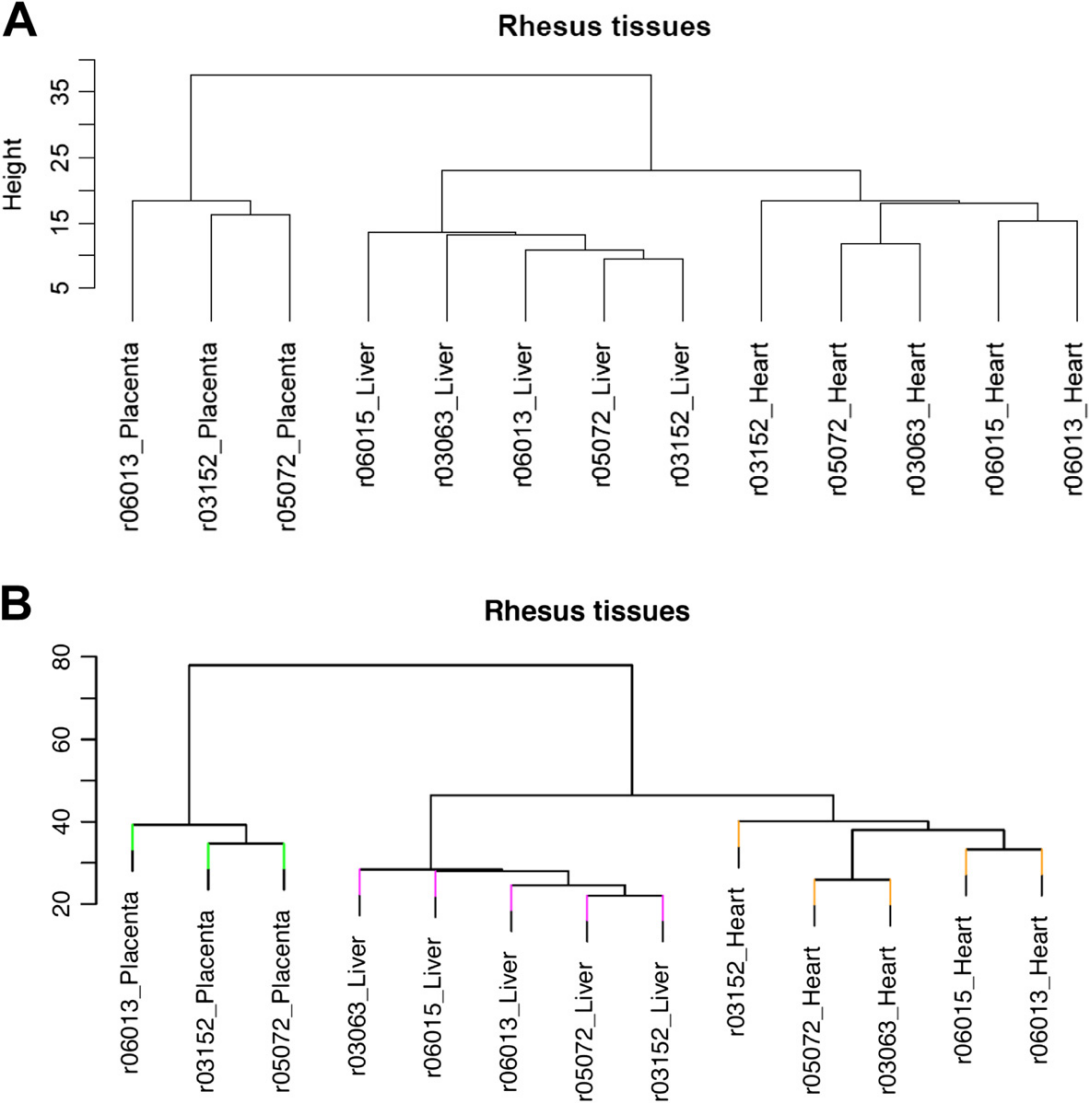
**Rhesus-competent probes distinguish monkey tissues.** Unsupervised hierarchical cluster analyses of methylation data generated for placenta and fetal tissues are shown for exact match (**A**; N = 35,901) or mismatch (**B**; N = 154,030) rhesus-competent probes. The length of the branches using the scale shown indicates relatedness.

Using only the rhesus-competent probes, we tested the reproducibility of this array on monkey DNAs extracted from skin biopsies of two healthy monkeys and found that each monkey’s replicates (N = 3) were highly correlated to each other regardless of the rhesus probe set (exact match or mismatch), with R^2^-values similar to those found among human replicates for this assay (R^2^ > 0.99; Figure 1A-B). Notably, the methylation levels of the rhesus probes were significantly correlated to the levels found in humans (Figure 1D). Together, these data suggested that both rhesus-competent probe sets within this assay are capable of providing an accurate quantitative measure of DNA methylation throughout the rhesus genome. As a further test of this conclusion, we used only rhesus-competent probes with data passing stringent quality control (exact match: N = 35,750; mismatch: N = 148,850; Methods) and investigated whether the rhesus-competent probes were capable of distinguishing between placental and fetal (heart and liver) tissues from five pregnancies. This analysis revealed, regardless of probe set, a clear hierarchical distinction of placenta and fetal tissues, as well as between the two fetal tissues (Figure 2), indicating that the rhesus-competent probes have robust tissue-distinguishing properties similar to those found when the full set of array probes are used in human studies [17]. Finding that both rhesus-competent probe sets generated correlations > 0.99 and tissue distinguishing hierarchical clusters that were nearly identical (Figure 2) suggested that the use of both sets was redundant. While we conducted all subsequent analyses with both probe sets and found nearly identical results, we only present the data from the more comprehensive set of rhesus-competent probes, the mismatch set (N = 148,850).

### Genome-wide DNA methylation analysis in rhesus macaque

**Figure 3:**
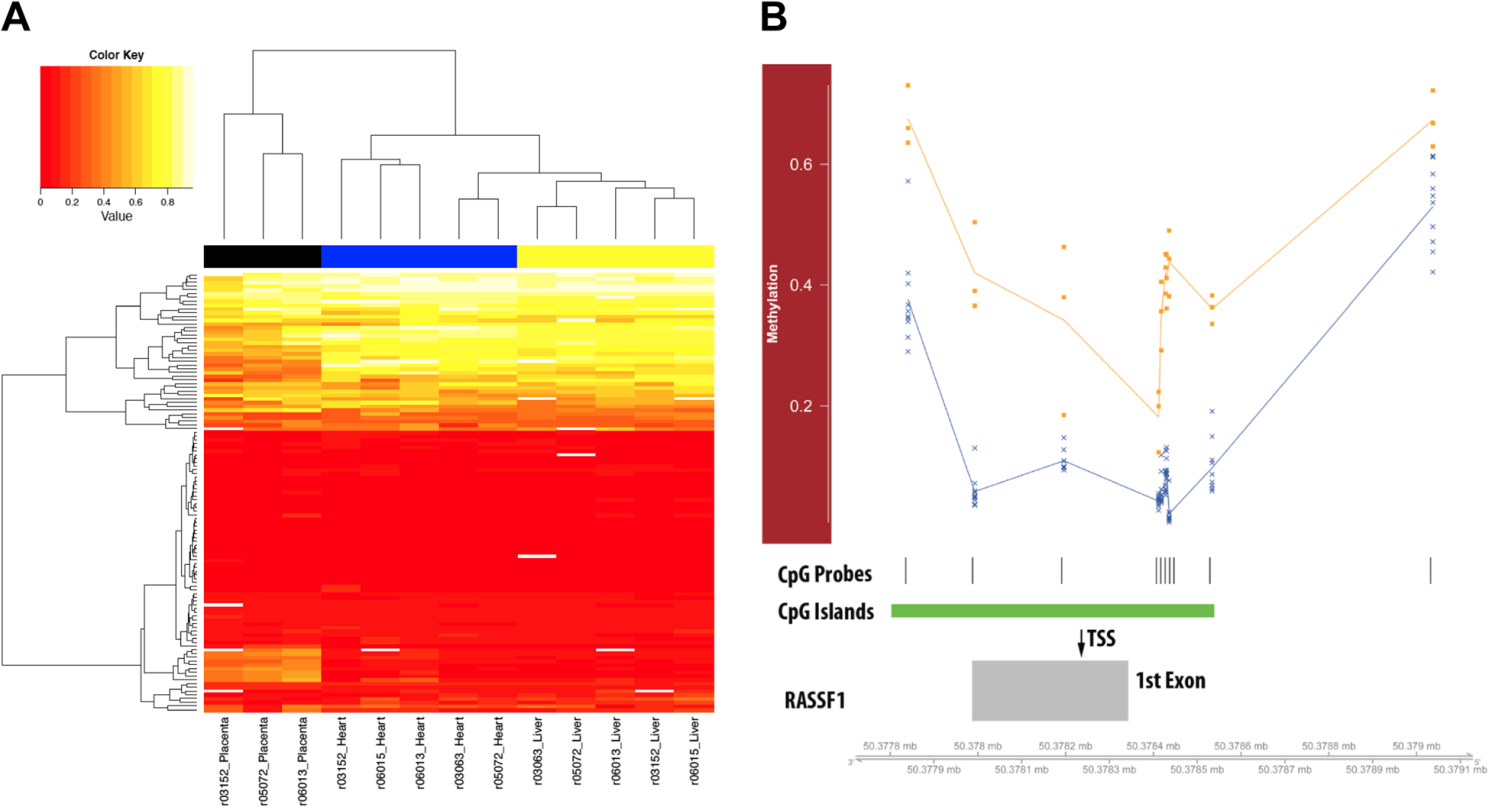
**Cancer-related loci distinguish tissues due to significant differential methylation. (A)** Unsupervised hierarchical cluster and heatmap of methylation levels at a limited set of cancer-loci previously investigated in placental tissue by Chui *et al.*[23]. The cluster tree indicates the relatedness of the three tissues (x-axis) or the methylation levels (y-axis). The heatmap uses a color scale to indicate the relative methylation level at each locus. Each row of colored lines (N = 117) represents the methylation level for each CpG locus: red for hypomethylated and yellow for hypermethylated (see legend). Each column (n = 13) corresponds to each tissue. **(B)** Relative locations of *RASSF1* methylation. Top panel shows the methylation levels (y-axis) in placenta (orange squares) and fetal (blue crosses) tissues at several loci annotated in or near the *RASSF1* locus. Shown below are the relative CpG coverage (vertical black lines) and CpG island location (green rectangle). Bottom panel depicts a partial gene schematic of *RASSF1*, indicating the relative location of the *RASSF1* transcription start site (TSS) and exon 1. The genomic position shown below is relative to HG-19 coordinates.

Placental tissue has drawn biological and gene expression analogies to malignant tissues [18-22]. A recent study used the traditional DNA methylation detection method (locus-specific sodium bisulfite sequencing) to compare the promoter regions of nine tumor suppressor genes in placental and fetal tissue and only found hypermethylation in the promoter of the Ras association domain family 1 A (*RASSF1A*) gene [23]. We determined that there are several rhesus-competent probes (N = 117) residing throughout the nine tumor suppressor genes interrogated by Chiu *et al*. (Additional file 3) and that these probes were able to distinguish between all three tissue types, particularly the fetal from the placental tissues (Figure 3A). Thus, we conducted a linear mixed-effects regression analysis (see Methods) on this restricted set of probes and found differential methylation throughout RASSF1 (N = 10; Figure 3B and Additional file 3) and several of the other tumor suppressor genes previously tested (MGMT (N = 7), DAPK1 (N = 3), and CDKN2B (N = 2); Additional file 3). Indeed, this assay simultaneously interrogates additional regions that were not investigated by Chiu and colleagues, which underscores the more comprehensive potential of this assay for DNA methylation detection in the rhesus macaque genome. Independent analysis of sodium bisulfite converted DNA spanning the genomic region interrogated by this assay corresponded well with the β values generated by the array (Additional file 2C-G), again validating the accuracy of this array and this set of probes to measure DNA methylation levels in the rhesus genome.

To further explore the potential of this assay, we used the rhesus-competent probes to conduct a genome-wide regression analysis of methylation levels between placental and fetal tissue and identified 6,102 differentially methylated loci (Bonferroni <0.05; Additional file 4). To statistically validate our methods and adjust for possible failure of asymptotic assumptions due to the small sample size, we performed one hundred permutations of the data and found that there was a high correlation between the asymptotic P-values and the permuted P-values (Additional file 2B and Additional file 4; Methods). Annotation of the differentially methylated loci using gene ontology and Kyoto encyclopedia of genes and genomes (KEGG) pathways revealed biologically relevant terms that were significantly overrepresented for developmental processes (Additional file 5). However, the KEGG analysis did not find an overrepresentation of cancer related terms, suggesting that normal tissue differentiation is the primary purpose of the different methylation marks. Nonetheless, we restricted the probes used in the regression analysis to those annotated to oncogenes and tumor suppressor genes and identified cancer-specific differentially methylated loci (N = 76; Additional file 6), suggesting placental tissue carries a cancer epigenetic signature.

### Species-specific methylation marks

These findings led us to test the hypothesis that other species-competent probe sets could be identified and used to study genome-wide DNA methylation. Thus we conducted a similar probe analysis to identify competent probes for the mouse (N = 9,734; Additional file 1) and the chimpanzee (N = 360,491; Additional file 1). Using the mouse-competent probes, we tested the reproducibility of this array and found that each mouse’s replicates (N = 3) were highly correlated to each other, with R^2^-values similar to those found among human and rhesus replicates for this assay (R^2^ > 0.99; Figure 1). In addition, using each set of species-competent probes (human, chimpanzee, rhesus, and mouse), we compared the consistency of this array between individuals of the same species (N = 3) and showed that there is a similarly high consistency among individuals of the same species (mean R^2^ > 0.90; Additional file 7), and that a set of common probes (N = 8,189) can clearly distinguish the species from one another (Additional file 8A). Furthermore, we investigated the gene structure distribution of the species-specific probes (i.e. monkey and mouse; Additional file 9). To test the significance of this probe distribution in monkey and mouse we compared the proportion of CpG loci that reside in each structure to the total number of CpG loci interrogated on the DNA methylation panel via permutation testing. We found that the optimization in both monkey and mouse is significantly biased (*P* < 0.0001) to retain more conserved gene structures (i.e. 5’ UTR, 1^st^ exon, and gene body regions). This finding is consistent with the probe selection method used to optimize the assay, which requires that the species-specific probes be a near match to the human array (allowing for only a four base mismatch), meaning that conserved genomic structures are going to be preferentially retained and that the analyses of non-human genomes are going to be limited to these regions. Together, these data suggest that this optimization method is generalizable to any sequenced mammalian species, making genome-wide DNA methylation detection possible for a much broader group of species.

### Novel utility of the array

**Figure 4:**
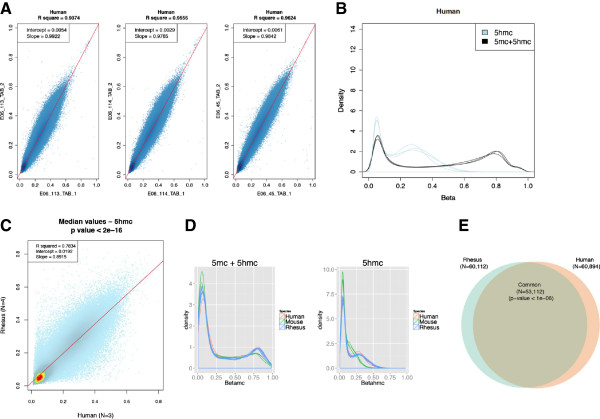
**Detection of *5-hmC *is highly reproducible and correlated among primates. A)** Scatter plots of human brain methylation data (*5-hmC*) generated from biological replicates run on the HumanMethylation450 BeadChips are shown to have a mean R^2^ > 0.95. Data for each probe is represented as a blue dot. For all scatter plots, the diagonal red line indicates the regression line and the x and y-axes indicate the methylation level for each replicate **(A)** or the median methylation values of the samples for each species **(C)**. **B)** The density (y-axis) of probes at each methylation level (x-axis; beta) in human brain samples (N = 3) that were either interrogated for total methylation (5mc + 5hmc; black line) or *5-hmC* levels (5hmc; blue line). **C)** Scatter plot showing the *5-hmC* correlations between rhesus (y-axis) and human (x-axis) data. Each probe is represented as a colored dot that are mostly light blue but become dark blue, green, yellow, or red as the density of loci increases. **D)** The density (y-axis) of probes at each methylation level (x-axis; beta) in human (red; N = 3), rhesus (blue; N = 7), and mouse (green; N = 3) brain samples that were either interrogated for total methylation (5mc + 5hmc; left plot) or *5-hmC* levels (5hmc; right plot). **E)** Venn diagram showing the overlap of the human and rhesus probes with a median *5-hmC* level > 20%.

The recent characterization of 5-hydroxymethylcytosine (*5-hmC*) in both stem cells and post-mitotic neurons, has led to a flurry of studies aimed to determine the role of this base in mediating epigenetic effects [8,24-28]. While numerous recent methods have emerged that are capable of providing base-resolution of *5-hmC*, these methods are sequencing-based, making them laborious and cost prohibitive [10,11]. Thus, we sought to determine if the aforementioned human DNA methylation array (HumanMethylation450 BeadChips; Illumina), which is designed to detect *5-mC*, could also report *5-hmC* levels throughout the genome. To test this hypothesis, we treated human brain DNA with β-glucosyltransferase and recombinant mouse Tet1 (mTET), prior to following the standard array protocol (Methods), and found that replicates (N = 3) were highly correlated to each other, with R^2^-values similar to those found for *5-mC* detection among human replicates (mean R^2^ > 0.95; Figure 4A). In comparison to the *5-mC* levels in these samples (median = 53%), the median *5-hmC* levels are much lower (21%), as the majority of the loci are < 40% 5’-hydroxymethylated (Figure 4B). Since independent analysis of *5-hmC* (Methods) in these tissues validated the accuracy of the assay (Additional file 2H-M), these data suggest that this array is capable of providing a quantitative measure of *5-hmC* throughout the human genome.

This finding prompted us to investigate whether the array could accurately detect *5-hmC* in other mammalian genomes and we found that it provides a consistent quantitative measure of *5-hmC* throughout both the monkey and mouse brain genomes (Additional file 8C-D); again suggesting that optimizing this assay is generalizable to any sequenced mammalian species. Comparison with the human *5-hmC* profiles also revealed several interesting biological findings, including that *5-hmC* profiles clearly distinguish between species (N = 8,189 probes; Additional file 8B) and that the primate median *5-hmC* levels are highly correlated (Figure 4C). Thus, despite the fact that there are species-distinguishing *5-hmC* marks, the majority of marks have a consistent level of *5-hmC*, at least in primates (Figure 4D), suggesting that there is likely to be a significant overlap of primate genes with abundant *5-hmC* levels. Consistent with this rationale, we found that primates indeed have a significant overlap of probes with > 20% *5-hmC* levels (Figure 4E). In addition, primates have significantly more probes with > 20% *5-hmC* than mice (53% (human), 42% (monkey), and 17% (mouse); Figure 4D), suggesting that this mark may play a larger role in primates than mice. Together, these data show that this array-based assay has a broad utility in DNA methylation studies of both *5-mC* and *5-hmC*.

### Hydroxymethylation and genomic context

**Figure 5:**
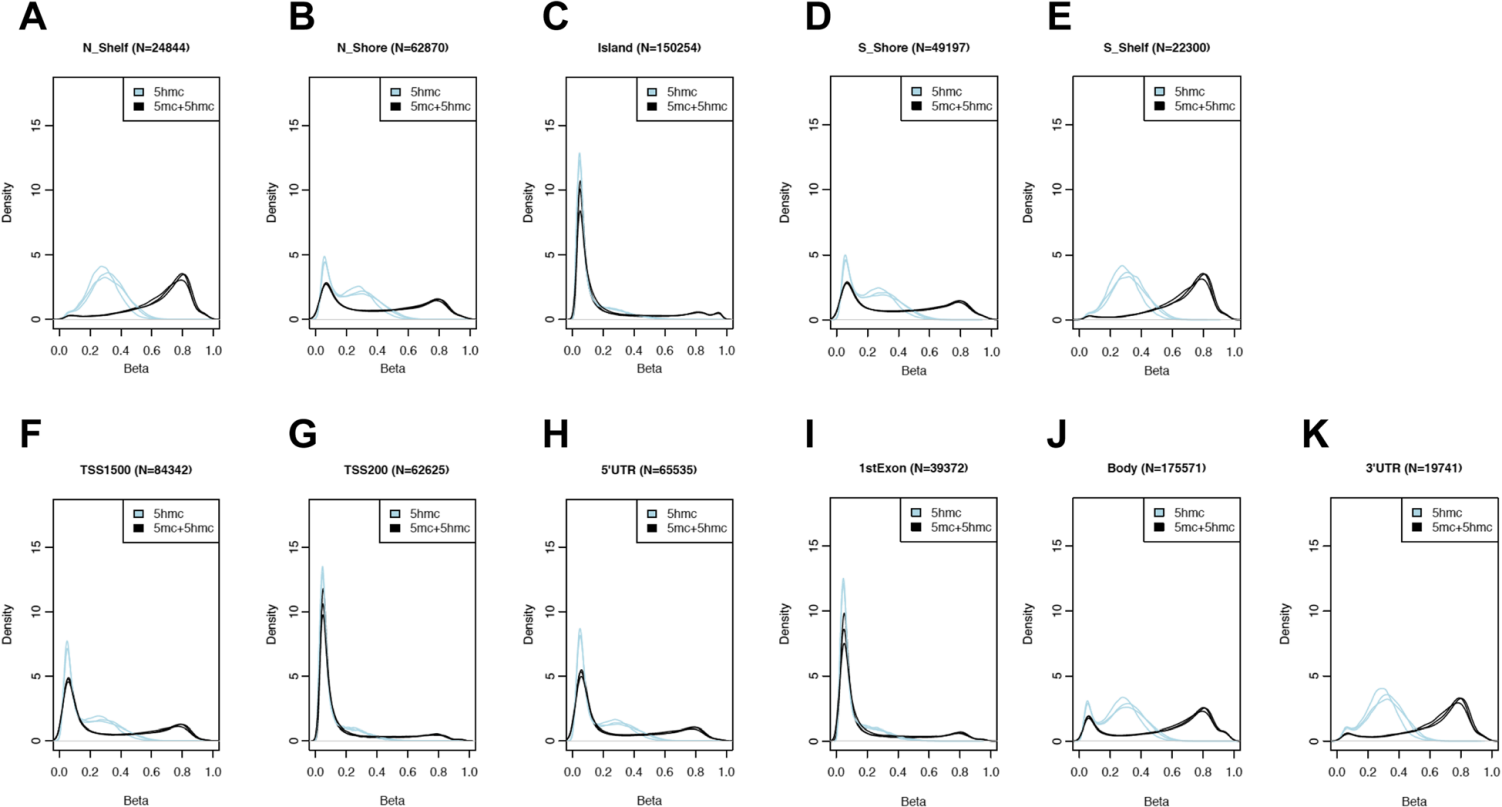
**The distribution of DNA methylation with respect to CpG islands and gene structures. A-K)** The density (y-axis) of probes at each methylation level (x-axis; beta) in human brain samples that were either interrogated for total methylation (5mc + 5hmc (black line)) or *5-hmC* levels (5hmc (blue line)). **A-E)** Profiles shown are delineated for the island, the island shores (N_shore (5’ end) and S_shore (3’ end)) or the island shelves (N_Shelf and S_Shelf, which are defined as 0-2 or 2-4 kilobases flanking the island, respectively. **F-I)** Profiles shown are delineated relative to a gene, including the distance to the gene transcription start site (TSS; TSS1500 (i.e. within 1500 bp of the TSS) and TSS200), 5’UTR, 1^st^ exon, body, and 3’UTR. The number of probes is indicated for each island and gene category.

The locations of methylated cytosines are often referred to with respect to either a CpG island or a gene. In regards to CpG islands, methylation is either on the island, on the island shores or on the island shelves (0-2 or 2-4 kilobases flanking the island, respectively; [29]). Recent studies of *5-hmC* in human embryonic stem cells (hESCs) have been contradictory regarding the *5-hmC* level on CpG islands, with the most recent report showing that CpG islands are deficient of *5-hmC*[10,11,25,26,30,31]. However, studies of *5-hmC* levels in mouse hippocampal and cerebellum tissue revealed that the prevalence of *5-hmC* on a CpG island is determined in relation to the nearest gene, meaning *5-hmC* is depleted on islands associated with a transcription start site (TSS) but enriched on intragenic islands [8]. To determine the distribution of *5-hmC* relative to CpG islands in our human, monkey and mouse brain tissues we compared the proportion of CpG loci with > 20% *5-hmC* that reside in or around CpG islands to the total number of CpG loci interrogated on the DNA methylation panel via permutation testing (see Methods). We found that all species have a significant overrepresentation of *5-hmC* on the island shelves (*P < 0.0001*) and a significant underrepresentation on the islands (*P < 0.0001*; Figure 5A-E, Additional file 10 and Additional file 11). However, when the CpGs on islands were segregated into associated or not associated with a TSS, we found in all species that probes in islands associated with a TSS have a lower *5-hmC* level (*P < 0.0001*)*.* Together, these data show a consistent genome distribution of *5-hmC* among species and support previous data from *hESCs* and mouse brain tissue, further validating this array for studies of *5-hmC*.

Locations of DNA methylation relative to a gene refer to regions that include the distance to the gene TSS (TSS1500 (i.e. within 1500 bp of the TSS) and TSS200), 5’UTR, 1^st^ exon, body, and 3’UTR. Previous studies of *5-hmC* levels in mouse hippocampal and cerebellum tissue revealed that *5-hmC* is enriched in gene bodies [8]. Here we found a similar enrichment in the gene body of all species (*P < 0.0001*) but our data also revealed that the 3’ UTR has more *5-hmC* than would be expected by chance alone (*P < 0.0001*; Figure 5F-K and Additional file 12). Since we also found a significant underrepresentation in all other genic regions (*P < 0.0001*), these data suggest that a non-stochastic mechanism may prevent *5-hmC* from the 5’ end of genes.

**Figure 6:**
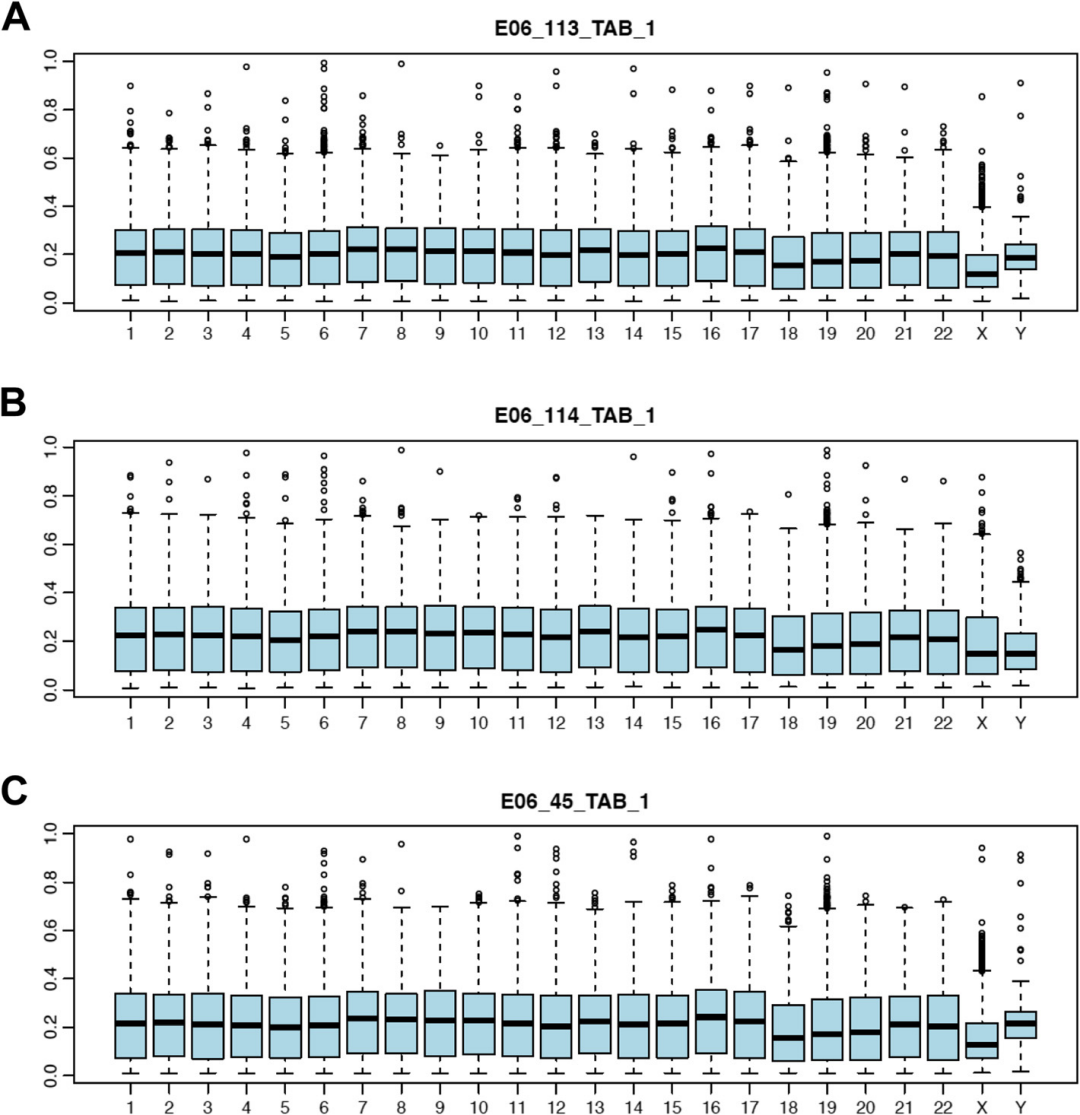
**The range of *5-hmC *levels on each chromosome.** The distribution of *5-hmC* in human brain tissues (N = 3; **A-C**) is shown using box and whisker plots depicting the *5-hmC* level (y-axis) of probes (N = 485,577) by chromosome (x-axis).

It is well known that specific regions of the genome are differentially methylated based on the biological functions of the genes contained within the region, with the most striking example being X inactivation in females. To determine if *5-hmC* is specific to certain regions of the genome, we plotted the genomic locations of the *5-hmC* and found that all three species have *5-hmC* levels greater than 20% located throughout the genome, but closer examination suggest a deficit of *5-hmC* on the X chromosome (Figure 6 and Additional file 13). To test the significance of these observations and to determine if other chromosomes contain a disproportionate number of *5-hmC* loci with levels > 20%, we compared the proportions of these *5-hmC* loci for each chromosome using permutation testing (see Methods) and found that only humans have chromosomes with a disproportionate number of loci containing *5-hmC* levels > 20%, both with deficits (X (*P* < 0.0001) and 18 (*P* < 0.03); Figure 6 and Additional file 11). Although the deficit on the X chromosome approaches significance in monkeys (*P* < 0.091), these data suggest that chromosomal deficits are human specific and that human chromosomes X and 18 are not governed by the same *5-hmC* kinetics as the rest of the genome.

### X-linked 5-hmC loci have functions in chromatin modifications and cell death

In humans the X chromosome was deficient of loci with high levels of *5-hmC.* Annotation of X-linked CpG loci with > 40% *5-hmC* in primates or > 20% in mice using gene ontology revealed several biologically relevant ontologies that were significantly overrepresented among all species. These findings predominately included terms associated with epigenetic functions including covalent chromatin modifications and histone acetylation. (Additional file 5; see Methods). Together, these data suggest that *5-hmC* may play a role in an epigenetic feedback loop*.* Interestingly, annotation of the human X-linked CpG loci with > 60% *5-hmC* using gene ontology revealed several biologically relevant ontologies that were significantly overrepresented for cell death and apoptotic processes, suggesting that *5-hmC* may target a specific subset of genes on the X chromosome. Notably, gene ontological analysis of the entire array (N = 21,231 genes) also finds significant terms, suggesting that the full array may have a bias toward a subset of genes and that conclusions from ontological analysis should be taken with caution. Clearly further studies are needed to determine the diverse functional contributions of *5-hmC* in the mammalian brain.

## Discussion

This study optimizes an established human array-based DNA methylation (*5-mC*) assay to allow for accurate detection of *5-mC* in non-human primates and mice and, perhaps more importantly, for the detection of genome-wide *5-hmC* in mammals. Optimization of this assay dramatically improves its utility because now it can be used to simultaneously investigate genome-wide *5-mC* and *5-hmC* in any sequenced mammalian genome. Moreover, since the analysis of data generated by this array is well established, this assay will reduce the cost and time of DNA methylation studies, allowing for a greater utilization of mammalian model systems to investigate the role of epigenetics in mammalian biological processes.

This optimization achieved high reproducibility among *5-mC* data generated for chimpanzee, rhesus, and mouse by utilizing an approach that relied on filtering the data down to the probes with a near match (allowing for up to 4 mismatches) between the human genome and the species of interest. This filter for species-specific probes still provides data for > 90% of the human annotated genes in the monkey and nearly 25% for the mouse. Finding that the chimpanzee analysis is able to retain far more probes (N = 360,491) is consistent with known evolutionary similarity with humans but also may reflect the higher quality reference sequence of the human and chimpanzee genomes compared to the rhesus genome. Recently, a similar approach identified 13,715 mouse-competent probes on the human array [32], which contains nearly 75% of the mouse-competent probes reported here. Together these independent findings strongly support the use of this array with other species. The only other array-based method available for *5-mC* detection in mice (Mouse CpG Island Microarray; AgilentTechnologies) relies heavily on the efficiency/reproducibility of restriction enzymes and the data is only reports *5-mC* methylation at CpG islands, which our data here clearly shows is lacking the majority of the variable DNA methylation information (Figure 5).

Having the ability to conduct an epigenome study in wide variety of sequenced mammals represents a significant new methodology. Here we compared the output of the optimized genome-wide array to that of a previously reported locus-specific study by Chiu et al., both investigating the epigenetic differences between monkey placenta and fetal tissue. Clearly the sheer magnitude of data from the array is not comparable between studies and neither are the conclusions. Notably, in 2007, when the Chui et al. study was conducted, the HumanMethylation450 BeadChips had not been developed; meaning Chiu and colleagues were using the molecular tools available at that time. This fact notwithstanding, while Chui et al. only found methylation differences in one of the nine interrogated genes (*RASSF1*), the much more comprehensive array approach developed here found differences in four out of the nine genes. In addition, the array identified differences in a total of 2,825 genes throughout the genome, including hypermethylated in cancer 1 (*HIC1*) and TIMP metallopeptidase inhibitor 3 (*TIMP3*), both of which were previously shown, using a locus-specific approach, to not be differentially methylated in normal placental tissue [33]. Together, these data highlight the dramatic advantage to using the array-based approach and suggest that studies of placental methylation need to be revisited.

It was previously shown that β-glucosyltransferase could be used to modify an existing array-based protocol for the detection of *5-hmC*[34]. However, in that case the array was not specifically designed for methylation detection, the method relied on the efficiency/reproducibility of restriction enzymes, and the data was biased toward CpG islands. In contrast, using a modified protocol and the HumanMethylation450 BeadChip largely overcomes these limitations and produces a more reliable unbiased profile of *5-hmC* throughout the genome, providing a new resource for epigenetic studies.

The density of *5-hmC* probes in all three species profiles suggests that the bulk of *5-hmC* levels are from 20% - 60%, especially for primates (Figure 4D). While it was expected that the majority of probes would have a low level of *5-hmC* (< 40%), we did not anticipate that, in the brain, nearly 50% of the primate loci would be > 20% hydroxymethylated and nearly 20% of human loci would be > 50% hydroxymethylated. These data suggest that *5-hmC* is much more prevalent than previously reported and that it may play a more significant role in primates than mice. However, the gene ontological analysis suggests that this role is in a feedback loop for epigenetic processes such as histone lysine methylation and that these functions are common to all mammals.

In the brain tissues of all three species we found the distribution of *5-hmC* with relation to gene structure to be consistent with previous reports that found *5-hmC* is especially prevalent in the regions adjacent to CpG islands (in the shelves), indicating that the highest levels of *5-hmC* are at regions of the genome with the lowest CpG density. Also consistent with previous work was the finding that *5-hmC* is more prevalent in gene bodies compared to the 5’ end of a gene; however, we find that the 3’ UTR is particularly enriched for this mark, a location that may facilitate a proposed role in a epigenetic feedback loop. At the chromosomal level *5-hmC* was reported elsewhere to be deficient from the X chromosome in mouse hippocampal and cerebellum tissue. In contrast, we didn’t find a deficiency on any mouse chromosomes but we did find it is significantly deficient on the human X and approaching significance on the monkey X. Although the synteny between human and mouse X chromosomes is high, the smaller number of loci from the mouse may have resulted in a higher genome-wide variance that precluded the mouse X data to reach significance.

As we learned with genome-wide association studies, successful epigenome-wide association studies of complex phenotypes are going to require the analysis of a very large number of individuals (N > 1000). The development of this assay improves our ability to quickly and reproducibly analyze the dynamic role of DNA methylation in a large cohort of individuals. This assay also augments the use of model systems for the study of epigenetic mechanisms and phenomena that are difficult to study in humans, including other recently identified modifications to methylcytosine, namely 5-formylcytosine and 5-carboxylcytosine. In addition, data from model systems will make it quite tempting to draw evolutionary conclusions between the different mammalian species and the gene ontology analysis shown here suggests that there are more similarities among species than differences. In short, this optimized assay holds great promise to improve our understanding of DNA methylation and its contributions to health, disease, and evolution.

## Conclusions

Here we have developed an array-based assay to detect *5-mC* and *5-hmC* in mammalian genomes and show that it is generalizable to all mammals. Using this assay we find that placental tissue carries a cancer epigenetic signature, that *5-hmC* has a hierarchical abundance among mammals, and that X-linked loci and the 3’UTR of genes have a heightened prevalence for *5-hmC*. In short, this optimized assay greatly improves the utility of mammalian model systems for studying the role of epigenetics in human health, disease, and evolution.

## Methods

### Tissue acquisition and DNA extraction

Except for the mouse brain tissues (UW IACUC protocol #M02529), all other tissues were supplied by other research groups, including monkey skin and brain tissue (Dr. Kalin’s group; UW IACUC protocol #G00181), monkey placental and fetal tissue (Dr. Golos’ group; UW IACUC protocol #G00245), and human brain tissue (Emory Neuroscience NINDS Core Facilities; approved by the Emory Internal Review Board on August 18, 2013 and given the approval number CR2_IRB00045782). All groups follow animal housing and experimental procedures that are in accordance with institutional guidelines. Once acquired, approximately thirty milligrams of tissue was homogenized with glass beads (Sigma) and DNA extraction was performed using AllPrep DNA/RNA mini kit (Qiagen).

### DNA methylation profiling

Genomic DNA was split into the following two aliquots: a sodium bisulfite-only (SB-only) aliquot; and a Tet1 treated followed by SB treated aliquot (TAB). The Tet1 treatment was conducted using previously described methods [11]. Briefly, one microgram (μg) of genomic DNA was spiked with four nanograms (ng) of synthetically methylated control DNAs (N = 3). Together, these DNAs were sonicated to 400 basepairs (bp) using a Diagenode Bioruptor Plus (Diagenode) and concentrated to 19 μl (Amicon Ultacel 100 k, Millipore). To mark *5-hmC*, the concentrated DNA was then glucosylated with T4-β-glucosyltransferase (1 μM; Wisegene) and UDP-glucose (200 μM, Wisegene) at 37°C for 1 hour, and purified using the QIAquick Nucleotide Removal Kit (Qiagen). To mark *5-mC*, five hundred ng of the glucosylated DNA was oxidized in the presence of recombinant mouse Tet1 (0.48 μg/μl; Wisegene) at 37°C for 1 hour followed by enzyme inactivation with protease K at 50°C for 1 hour. The oxidized DNA was twice purified (Micro Bio-Spin 30, Bio-Rad; QIAquick PCR Purification Kit, Qiagen) and eluted in 20 μl of water. SB-treatment of both aliquots: Five hundred nanograms of genomic DNA or Tet-treated DNA was sodium bisulfite–treated for cytosine (C) to thymine (T) conversion using the EZ DNA Methylation-Gold kit (Zymo Research). The converted DNA was purified and prepped for analysis on the Illumina HumanMethylation450 BeadChips following the manufacturer’s guidelines. Briefly, converted DNA was amplified, fragmented, and hybridized to the HumanMethylation450 pool of allele-differentiating oligonucleotides. After a series of extension, ligation, and cleanup reactions, the DNA was labeled with a fluorescent dye. The labeled DNA was then scanned using an Illumina BeadArray Reader or iScan. Image analysis and signal determination were performed using the GenomeStudio software, Methylation Module (Illumina).

### Interpretation and QC of DNA methylation data

CpG DNA methylation data were interpreted using GenomeStudio to quantify methylated (M) and unmethylated (U) signal intensities for genomic DNA. The signals were quantile normalized, and overall methylation levels (β) were calculated as the ratio of methylated to total signal [i.e. β = M/(M + U + 100)], where β ranges from 0 (unmethylated) to 1 (methylated). For human samples, quality control of data resulted in removal of samples with aberrantly low signal intensity (mean < 2000) or with fewer than 99% of CpG loci detected, where a given locus was deemed not detected if the detection P-value was > 0.01 (detection P-value provided by Genome Studio and calculated relative to background signal). For rhesus, chimp and mouse samples, the data for only the ‘competent’ probe-set (ref. section ‘Assay optimization’) for each species was taken. Furthermore, any probes having more than 20% of samples with detected P-values > 0.01 were discarded from the analysis. Assay controls were inspected to remove samples with poor bisulfite conversion, staining, extension (single nucleotide extension assay), hybridization, or specificity. Outliers identified by hierarchical clustering and/or dissimilarity matrices were removed. Additionally, one control DNA replicate was run on each BeadChip to assess overall assay reproducibility. Methylation profiles of the control DNA correlated well, with an average Pearson correlation coefficient (R) of 0.990 between human replicates.

### Analysis of cancer-associated CpG loci

To analyze DNA methylation differences associated with cancer, we fit a separate regression for each CpG site. Although samples were randomly distributed across BeadChips and experiments with respect to disease, BeadChip was also included as a random effect covariate in all analyses to account for potential batch effects. The package “nlme” in R (Cran) was used for the mixed effect model. Fixed effects included intensity in the model. To correct for multiple hypothesis testing, we applied a Benjamini-Hochberg False Discovery Rate (FDR) correction using the R function “p.adjust,” but to avoid false positives due to the small sample size, we used conservative Bonferroni adjustment for our ultimate determination of significance.

### Permutation analyses for rhesus differentially methylated loci

All permutation analyses were conducted in R using the same linear model as the actual analysis, where BeadChip was treated as a mixed-effects covariate, but in each permutation the tissue type of the sample was randomly reassigned. In total, 100 permutations were conducted. Permutation P-values for each CpG locus were calculated by assessing the number of times each locus was more significantly associated with fetal tissue in the 100 permuted data sets than the actual.

### Analysis of genomic location

Loci were mapped to chromosome location using the Illumina annotation files for the HumanMethylation450 panel based on the NCBI Human Genome (build 37). Over- and underrepresentation of CpG loci with > 20% median *5-hmC* were assessed using both Fisher’s exact test and permutation analysis, which corresponded well (data not shown). Permutation *P*-values were determined as described previously [16], by comparing the proportion of >20% *5-hmC* loci on a chromosome (termed “actual proportion”) to the proportions obtained using the permuted loci (termed “permuted proportions”). To correct for multiple hypotheses (*i.e.* 24 chromosomes and both over and underrepresentation) the actual proportion of each chromosome was compared to the permuted proportions of any chromosome for each permutation. Thus the permutation *P*-value is the number of times a permuted proportion is more significant than the actual proportion, divided by the number of permutations (10,000).

### Data access

We have submitted the data generated from the monkey, chimp, human and mouse samples for this study to the Gene Expression Omnibus (GEO), which can be found under the Gene Series: GSE49177.

## Competing interests

The authors declare that they have no competing interests.

## Authors’ contributions

PC conceived of the study, analyzed the data and wrote the manuscript. LAP performed experiments and wrote the manuscript. ATJW and AH performed the validation experiments. RMB developed the software to display the validation data. PHR, MAG, and TGG selected and acquired samples. STW provided critical conceptual advice and the arrays, and helped write the manuscript. RSA conceived of the study, analyzed the data and wrote the manuscript. All authors read and approved the final manuscript.

## Supplementary Material

Additional file 1 **Optimized probe sets for each species.** The list of Illumina probe identification names that were used for each non-human species. Header indicates the species and whether these probes were identified as exact matches or mismatches (see Methods). Click here for file

Additional file 2 **Array-based assay probe distribution and validation.** A) The density (y-axis) of genes and the number of probes for each gene (x-axis; beta) are shown for human (blue), rhesus (orange), and mouse (black) genomes. B) Permutation analysis of placenta-associated differential methylation. Scatter plot of permuted (100 permutations) placental-associated *P*-values (x-axis) compared to asymptotic *P*-values (y-axis) calculated using the linear model (Pearson R = 0.9852). C-M) Independent assay validates the accuracy of the array for *5-mC* (rhesus) and *5-hmC* (human and rhesus) C-G) Bar charts showing the side-by-side comparison of the methylation levels (y-axis) of CpG loci near genes (denoted above each chart) in different monkey tissues that were determined by either sodium bisulfite treatment, cloning, and sequencing (white bars) or the HumanMethylation450 BeadChips (black bars). H-M) Circle plots showing the *5-mC* (H, J, L) and *5-hmC* (I, K, M) status of individual CpG loci in human (H-K) and monkey (L-M) genes (denoted above each plot) from brain tissue. Each circle represents the methylation status of an independent clone (open circle = unmethylated; closed circle = methylated). The numbers along the left of each panel indicates the percent methylation that was determined using either the HumanMethylation450 BeadChips (Array) or the independent assay (sodium-bisulfite sequencing; SBS). The numbers along the left indicates the nucleotide number from the transcription start site of each gene, as referenced from UCSC build 37/hg19. Each row of the panels indicates a different clone. Click here for file

Additional file 3 **Differentially methylated loci identified between rhesus placental and fetal tissue using genes previously investigated by Chui *et al. *** [23]**.** Header descriptions include: the Illumina assay probe identification (cgid); the Bonferroni corrected P-value (bonferroni); the false discovery rate (fdr); uncorrected P-value (none); permutation P-value (perm_pvalue); the genome build that the probe was designed on (Genome_Build); the human chromosome that the probe is located on (CHR); the genomic coordinates on the human chromosome (MAPINFO); the gene name as annotated by the University of California Santa Cruz genome browser (UCSC_RefGene_Name); the UCSC accession number for the gene (UCSC_RefGene_Accesssion); the location of the locus in relation to the UCSC gene structure (UCSC_RefGene_Group); and the location of the locus in relation to a UCSC annotated CpG island (Relation_to_UCSC_CpG_Island; blank means no association). Click here for file

Additional file 4 **Genome-wide differentially methylated loci identified between rhesus placental and fetal tissue.** Header descriptions include: the Illumina assay probe identification (cgid); the Bonferroni corrected P-value (bonferroni); the false discovery rate (fdr); uncorrected P-value (cls_pvalue); the genome build that the probe was designed on (Genome_Build); the human chromosome that the probe is located on (CHR); the genomic coordinates on the human chromosome (MAPINFO); the gene name as annotated by the University of California Santa Cruz genome browser (UCSC_RefGene_Name); the UCSC accession number for the gene (UCSC_RefGene_Accesssion); the location of the locus in relation to the UCSC gene structure (UCSC_RefGene_Group); and the location of the locus in relation to a UCSC annotated CpG island (Relation_to_UCSC_CpG_Island; blank means no association). Click here for file

Additional file 5 **Output results from gene ontology and KEGG analysis.** This table has five pages that can be accessed using the labeled tabs at the bottom. The first two tabs are gene ontology (DML_GeneOntology_Placenta_vs_Fe; tab 1) or KEGG analysis (DML_KEGG_Placenta_vs_Fetal.csv; tab 2) using the 6,102 loci identified with the genome-wide regression analysis. The header descriptions include: the gene ontology or KEGG identification number (GOBPID or KEGGID); the P-value associated with finding this term (Pvalue); the Odds ratio for the term (OddsRatio); the expected number of genes to be associated with the term (ExpCount); the actual number of the differentially methylated genes that are associated with the term (Count); the total number of genes on the assay associated with the term (Size); and the term name (Term). The other three pages of this file are the output results from a gene ontology analysis using the X-linked CpG loci with > 40% *5-hmC* in primates (5hmc_Chr_X_Human_GeneOntology_0; tab 3 (human) and 5hmc_Chr_X_Rhesus_GeneOntology_; tab 4 (monkey)) or > 20% in mice (5hmc_Chr_X_Mouse_GeneOntology_0; tab 5). Header descriptions are the same as above for tab 1. Click here for file

Additional file 6 **Differentially methylated loci identified between rhesus placental and fetal tissue using genes annotated to oncogenes and tumor suppressor genes.** Header descriptions include: the Illumina assay probe identification (cgid); the Bonferroni corrected P-value (bonferroni); the false discovery rate (fdr); uncorrected P-value (cls_pvalue); the genome build that the probe was designed on (Genome_Build); the human chromosome that the probe is located on (CHR); the genomic coordinates on the human chromosome (MAPINFO); the gene name as annotated by the University of California Santa Cruz genome browser (UCSC_RefGene_Name); the UCSC accession number for the gene (UCSC_RefGene_Accesssion); the location of the locus in relation to the UCSC gene structure (UCSC_RefGene_Group); and the location of the locus in relation to a UCSC annotated CpG island (Relation_to_UCSC_CpG_Island; blank means no association). Click here for file

Additional file 7 **Mammalian DNA methylation levels detected on the human array are highly correlated.** Scatter plots of human (A), chimpanzee (B), rhesus (C), and mouse (D) methylation data (*5-mC*) generated from biological replicates run on the HumanMethylation450 BeadChips are shown for mismatch species-competent probes (N = 485,577 (A); 360,491 (B); 154,030 (C), or 9,734 (D)). The diagonal red line indicates the regression line and the x and y-axes indicate the methylation level for each individual. The correlation level (R^2^) is denoted above each plot. Click here for file

Additional file 8 ***5-hmC *** **distinguishes species and is highly reproducible among mammals.** Unsupervised hierarchical cluster analyses of *5-mC* (A) and *5-hmC* (B) probe data common to all species (N = 8,189) that was generated from brain tissue of human (E06 samples), monkey (r# samples), and mouse (mA, mB, and mC) individuals are shown. The length of the branches using the scale shown indicates relatedness. Scatter plots of rhesus (C) and mouse (D) brain methylation data (*5-hmC*) generated from biological replicates run on the HumanMethylation450 BeadChips are shown to have a mean R^2^ > 0.92. Data for each probe is represented as a blue dot. For all scatter plots, the diagonal red line indicates the regression line and the x and y-axes indicate the methylation level for each replicate. Click here for file

Additional file 9 **Gene structure specific probe distribution among species.** The density (y-axis) of probes at each annotated gene structure (x-axis) using the human (blue), rhesus (orange), and mouse (black) optimization of the array. Click here for file

Additional file 10 **The distribution of DNA methylation with respect to CpG islands.** The density (y-axis) of probes at each methylation level (x-axis; beta) in monkey (A-E) and mouse (F-J) brain samples that were either interrogated for total methylation (5mc + 5hmc (black line)) or *5-hmC* levels (5hmc (blue line)). Profiles shown are delineated for the island, the island shores (N_shore (5’ end) and S_shore (3’ end)) or the island shelves (N_Shelf and S_Shelf, which are defined as 0-2 or 2-4 kilobases flanking the island, respectively. The number of probes is indicated for each island category. Click here for file

Additional file 11 **This table displays the output for the analysis of the locations of methylated cytosines with respect to either a CpG island or a gene.** This table has five pages that can be accessed using the labeled tabs at the bottom. The first three pages are with regard to CpG island and gene structures in human (5hmc_Human_Mismatch_pvalues_Reg; tab 1), monkey (5hmc_Rhesus_Mismatch_pvalues_Reg; tab 2), and mouse (5hmc_Mouse_Mismatch_pvalues_Reg; tab 3). The last three tabs are the results from our chromosomal analysis on human (5hmc_Human_Mismatch_pvalues_Chr; tab 4), monkey (5hmc_Rhesus_Mismatch_pvalues_Ch; tab 5), and mouse (5hmc_Mouse_Mismatch_pvalues_Chr; tab 6). All of the pages in this file have the following header descriptions: a genomic description of the locus location (Region (tabs 1-3); Chromsome (tabs 4-6)); whether the data is filter for probes > 20% or > 40% *5-hmC* (Cutoff); the total number of probes in the dataset (NumProbes_Dataset); the number of probes after the filter has been applied (NumProbes_Cutoff); the number of probes in the genomic description of interest (NumProbes_Region or NumProbes_Chr); the number of probes that meet the genomic description and the filter criteria (NumCommonProbes); the Fisher’s exact test P-value (FisherPvalue); the odd ratio for the result (OddsRatio); and the permuted P-value (PermPvalue). The tabs showing the chromosomal analysis also have a column that shows the variance that the data has for each chromosome (Variance). See text for definitions of the regions. Click here for file

Additional file 12 **The distribution of DNA methylation with respect to gene structures.** The density (y-axis) of probes at each methylation level (x-axis; beta) in monkey (A-F) and mouse (G-L) brain samples that were either interrogated for total methylation (5mc + 5hmc (black line)) or *5-hmC* levels (5hmc (blue line)). Profiles shown are delineated relative to a gene, including the distance to the gene transcription start site (TSS; TSS1500 (i.e. within 1500 bp of the TSS) and TSS200), 5’UTR, 1^st^ exon, body, and 3’UTR. The number of probes is indicated for each island and gene category. Click here for file

Additional file 13 **The range of *5-hmC *** **levels on each chromosome.** The distribution of *5-hmC* in monkey (N = 4; A-D) and mouse (N = 3; E-G) brain tissues is shown using box and whisker plots depicting the *5-hmC* level (y-axis) of probes by chromosome (x-axis). Click here for file
