# Supplementary material for: Array-based assay detects genome-wide 5-mC and 5-hmC in the brains of humans, non-human primates, and mice
Source: BMC Genomics. 2014 Feb 13;15:131. doi: 10.1186/1471-2164-15-131 (PMC3930898; doi:10.1186/1471-2164-15-131)
Supplement: Additional file 2 — Array-based assay probe distribution and validation. A) The density (y-axis) of genes and the number of probes for each gene (x-axis; beta) are shown for human (blue), rhesus (orange), and mouse (black) genomes. B) Permutation analysis of placenta-associated differential methylation. Scatter plot of permuted (100 permutations) placental-associated P-values (x-axis) compared to asymptotic P-values (y-axis) calculated using the linear model (Pearson R = 0.9852). C-M) Independent assay validates the accuracy of the array for 5-mC (rhesus) and 5-hmC (human and rhesus) C-G) Bar charts showing the side-by-side comparison of the methylation levels (y-axis) of CpG loci near genes (denoted above each chart) in different monkey tissues that were determined by either sodium bisulfite treatment, cloning, and sequencing (white bars) or the HumanMethylation450 BeadChips (black bars). H-M) Circle plots showing the 5-mC (H, J, L) and 5-hmC (I, K, M) status of individual CpG loci in human (H-K) and monkey (L-M) genes (denoted above each plot) from brain tissue. Each circle represents the methylation status of an independent clone (open circle = unmethylated; closed circle = methylated). The numbers along the left of each panel indicates the percent methylation that was determined using either the HumanMethylation450 BeadChips (Array) or the independent assay (sodium-bisulfite sequencing; SBS). The numbers along the left indicates the nucleotide number from the transcription start site of each gene, as referenced from UCSC build 37/hg19. Each row of the panels indicates a different clone. [file 1471-2164-15-131-S2.pptx]

## Slide 1
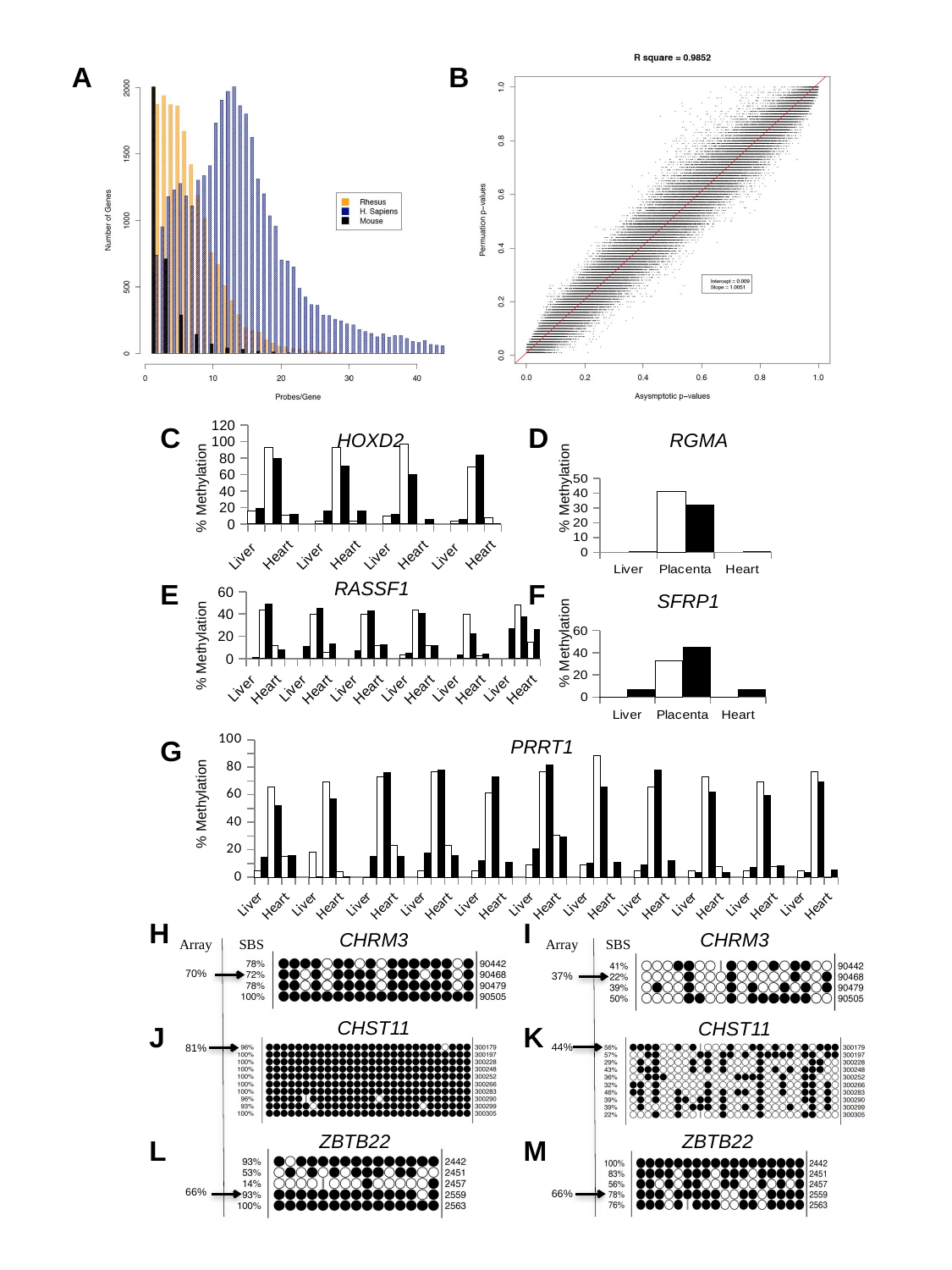

A
B
C
### Chart
| Category | | |
|---|---|---|
| Liver | 15.625 | 18.77 |
| Placenta | 93.10344827586205 | 80.0 |
| Heart | 10.71428571428571 | 11.77 |
| | None | None |
| Liver | 3.125 | 16.16 |
| Placenta | 93.10344827586205 | 70.0 |
| Heart | 3.571428571428571 | 15.85 |
| | None | None |
| Liver | 9.375 | 11.38 |
| Placenta | 96.55172413793096 | 60.0 |
| Heart | 0.0 | 6.02 |
| | None | None |
| Liver | 3.125 | 5.649999999999998 |
| Placenta | 68.9655172413794 | 84.0 |
| Heart | 7.142857142857141 | 1.02 |D
HOXD2
RGMA
% Methylation
% Methylation
### Chart
| Category | | |
|---|---|---|
| Liver | 0.0 | 1.0 |
| Placenta | 41.3793103448276 | 32.0 |
| Heart | 0.0 | 1.0 |
### Chart
| Category | | |
|---|---|---|
| Liver | 0.0 | 1.46 |
| Placenta | 44.0 | 49.01 |
| Heart | 11.76470588235294 | 7.619999999999996 |
| | None | None |
| Liver | 0.0 | 11.31 |
| Placenta | 40.0 | 45.0 |
| Heart | 5.882352941176467 | 13.11 |
| | None | None |
| Liver | 0.0 | 6.89 |
| Placenta | 40.0 | 42.91 |
| Heart | 11.76470588235294 | 12.7 |
| | None | None |
| Liver | 3.448275862068965 | 5.28 |
| Placenta | 44.0 | 40.53 |
| Heart | 11.76470588235294 | 11.86 |
| | None | None |
| Liver | 0.0 | 3.74 |
| Placenta | 40.0 | 22.34 |
| Heart | 2.941176470588235 | 4.37 |
| | None | None |
| Liver | 0.0 | 27.0 |
| Placenta | 48.0 | 37.75 |
| Heart | 14.70588235294118 | 26.72 |E
RASSF1
F
SFRP1
### Chart
| Category | | |
|---|---|---|
| Liver | 0.0 | 7.0 |
| Placenta | 32.25806451612903 | 44.9 |
| Heart | 0.0 | 6.9 |% Methylation
% Methylation
G
PRRT1
### Chart
| Category | | |
|---|---|---|
| Liver | 4.545454545454546 | 14.7 |
| Placenta | 65.3846153846154 | 52.3 |
| Heart | 15.38461538461538 | 15.7 |
| | None | None |
| Liver | 18.1818181818182 | 0.5 |
| Placenta | 69.23076923076921 | 57.2 |
| Heart | 3.846153846153844 | 0.4 |
| | None | None |
| Liver | 0.0 | 15.1 |
| Placenta | 73.07692307692305 | 76.3 |
| Heart | 23.07692307692307 | 14.9 |
| | None | None |
| Liver | 4.545454545454546 | 17.6 |
| Placenta | 76.92307692307686 | 78.3 |
| Heart | 23.07692307692307 | 15.9 |
| | None | None |
| Liver | 4.545454545454546 | 11.8 |
| Placenta | 61.53846153846154 | 73.3 |
| Heart | 0.0 | 10.6 |
| | None | None |
| Liver | 9.0909090909091 | 20.6 |
| Placenta | 76.92307692307686 | 81.6 |
| Heart | 30.76923076923077 | 29.3 |
| | None | None |
| Liver | 9.0909090909091 | 10.4 |
| Placenta | 88.46153846153847 | 65.4 |
| Heart | 0.0 | 10.6 |
| | None | None |
| Liver | 4.545454545454546 | 9.200000000000001 |
| Placenta | 65.3846153846154 | 77.7 |
| Heart | 0.0 | 11.7 |
| | None | None |
| Liver | 4.545454545454546 | 3.6 |
| Placenta | 73.07692307692305 | 62.0 |
| Heart | 7.692307692307692 | 3.1 |
| | None | None |
| Liver | 4.545454545454546 | 7.0 |
| Placenta | 69.23076923076921 | 59.2 |
| Heart | 7.692307692307692 | 8.6 |
| | None | None |
| Liver | 4.545454545454546 | 3.4 |
| Placenta | 76.92307692307686 | 69.6 |
| Heart | 0.0 | 5.3 |% Methylation
H
I
CHRM3
CHRM3
Array
SBS
Array
SBS
70%
37%
CHST11
CHST11
J
K
44%
81%
ZBTB22
ZBTB22
L
M
66%
66%
