# Supplementary figures and images for: Array-based assay detects genome-wide 5-mC and 5-hmC in the brains of humans, non-human primates, and mice
Source: BMC Genomics. 2014 Feb 13;15:131. doi: 10.1186/1471-2164-15-131 (PMC3930898; doi:10.1186/1471-2164-15-131)

## Slide 1
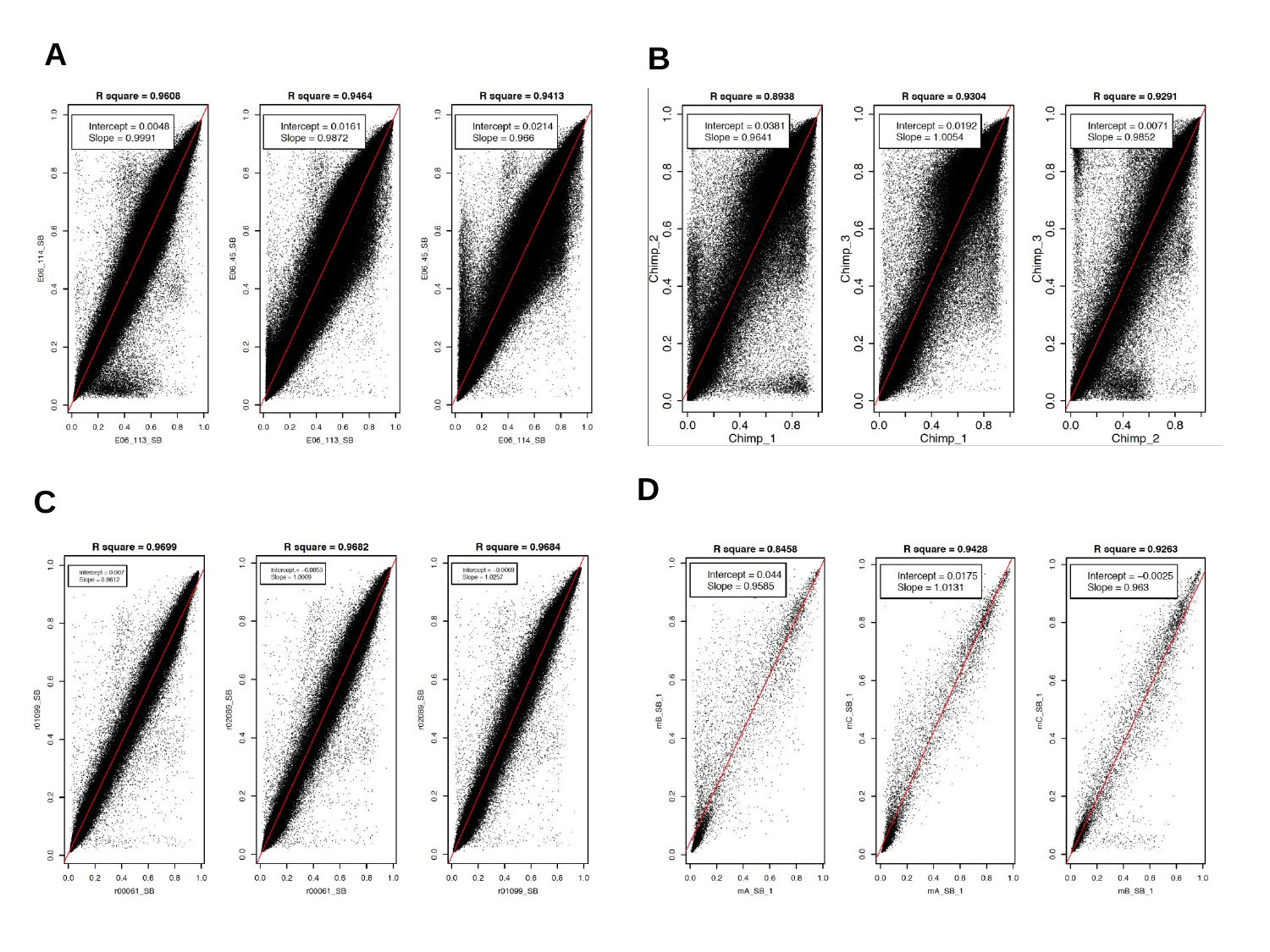

A
B
D
C

Supplement: Additional file 7 — Mammalian DNA methylation levels detected on the human array are highly correlated. Scatter plots of human (A), chimpanzee (B), rhesus (C), and mouse (D) methylation data (5-mC) generated from biological replicates run on the HumanMethylation450 BeadChips are shown for mismatch species-competent probes (N = 485,577 (A); 360,491 (B); 154,030 (C), or 9,734 (D)). The diagonal red line indicates the regression line and the x and y-axes indicate the methylation level for each individual. The correlation level (R2) is denoted above each plot. [file 1471-2164-15-131-S7.pptx]

## Slide 1
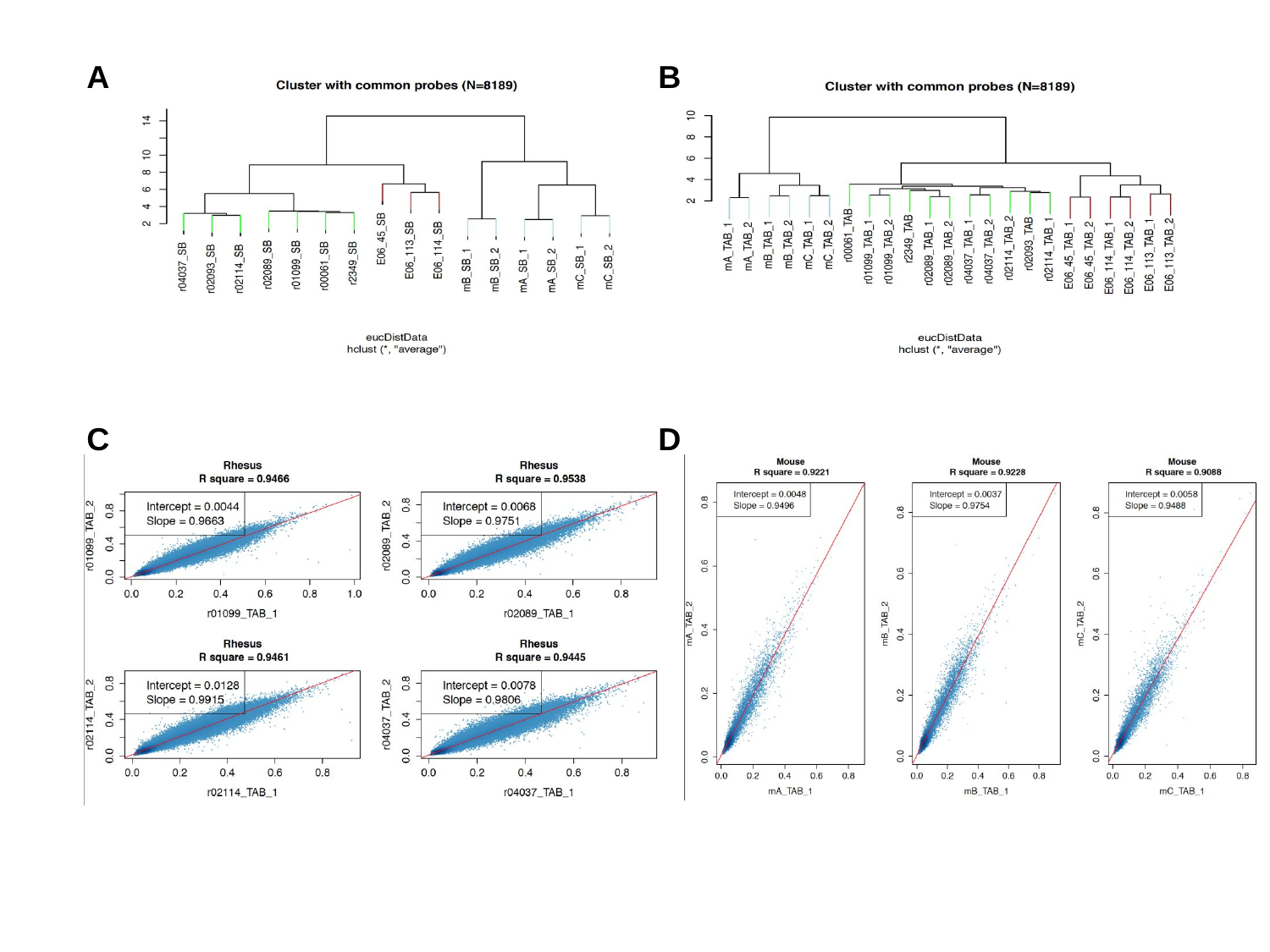

A
B
C
D

Supplement: Additional file 8 — 5-hmC distinguishes species and is highly reproducible among mammals. Unsupervised hierarchical cluster analyses of 5-mC (A) and 5-hmC (B) probe data common to all species (N = 8,189) that was generated from brain tissue of human (E06 samples), monkey (r# samples), and mouse (mA, mB, and mC) individuals are shown. The length of the branches using the scale shown indicates relatedness. Scatter plots of rhesus (C) and mouse (D) brain methylation data (5-hmC) generated from biological replicates run on the HumanMethylation450 BeadChips are shown to have a mean R2 > 0.92. Data for each probe is represented as a blue dot. For all scatter plots, the diagonal red line indicates the regression line and the x and y-axes indicate the methylation level for each replicate. [file 1471-2164-15-131-S8.pptx]

## Probe distribution

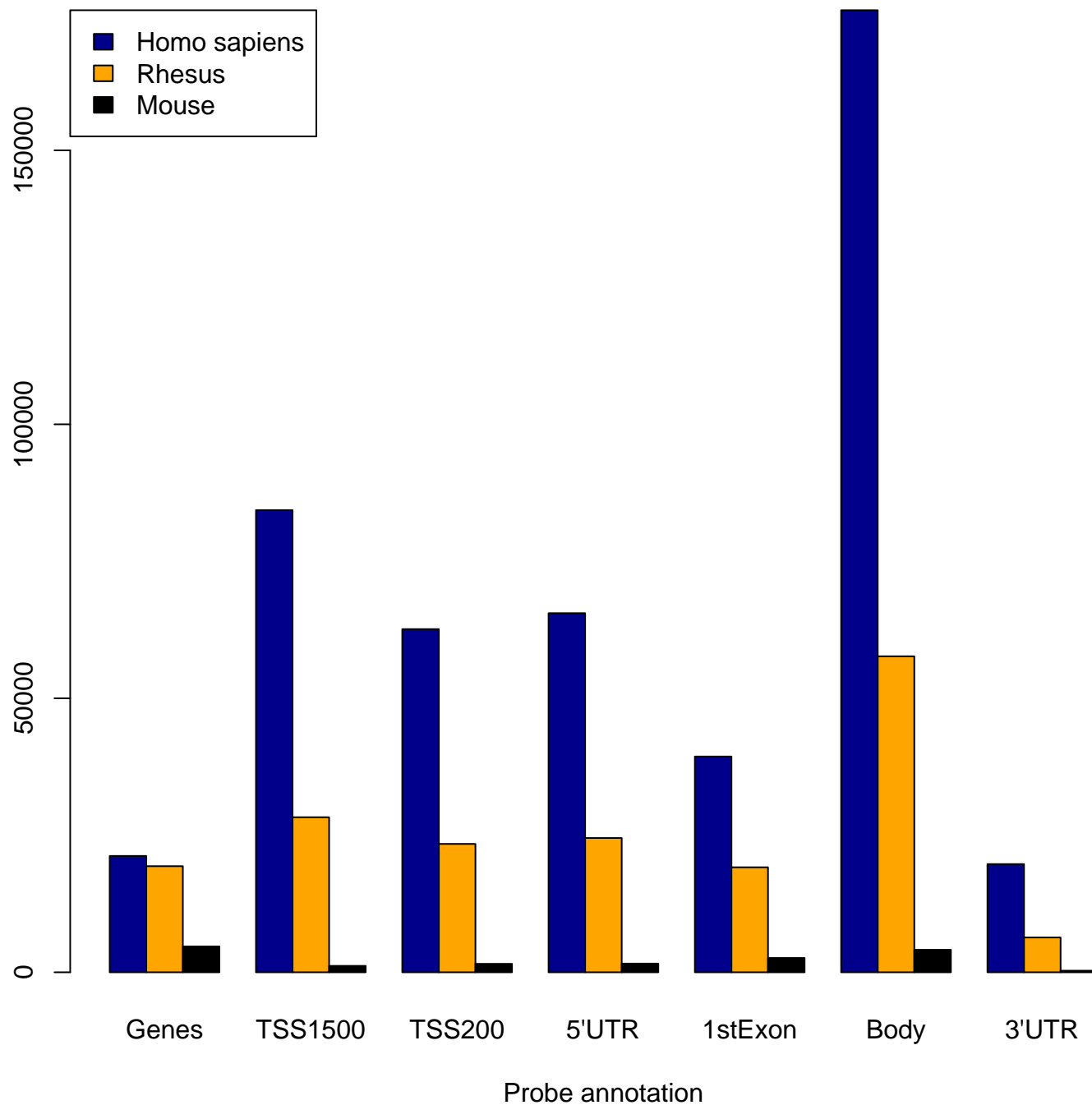

Supplement: Additional file 9 — Gene structure specific probe distribution among species. The density (y-axis) of probes at each annotated gene structure (x-axis) using the human (blue), rhesus (orange), and mouse (black) optimization of the array. [file 1471-2164-15-131-S9.pdf]

## Slide 1
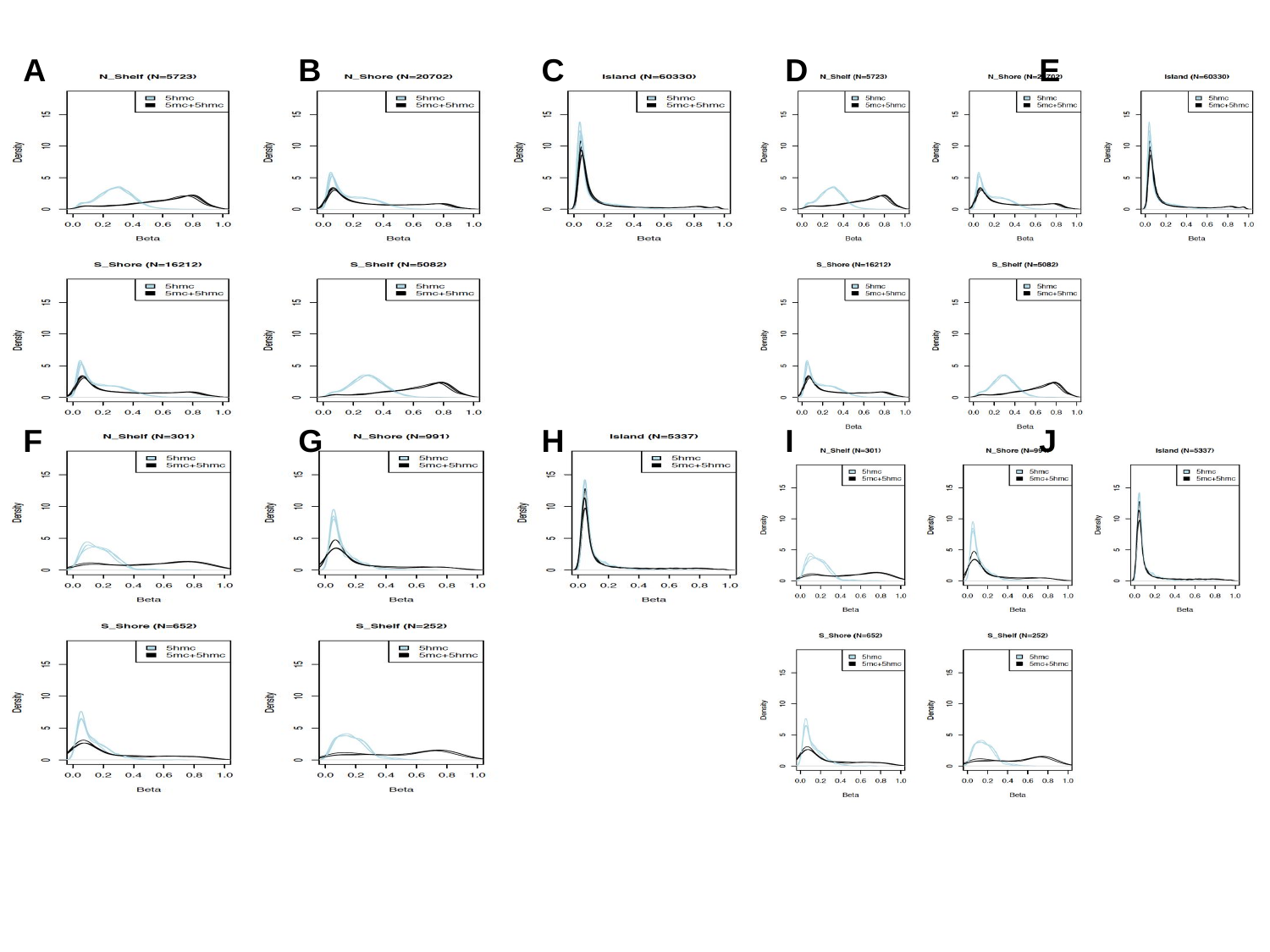

A
B
C
D
E
F
G
H
I
J

Supplement: Additional file 10 — The distribution of DNA methylation with respect to CpG islands. The density (y-axis) of probes at each methylation level (x-axis; beta) in monkey (A-E) and mouse (F-J) brain samples that were either interrogated for total methylation (5mc + 5hmc (black line)) or 5-hmC levels (5hmc (blue line)). Profiles shown are delineated for the island, the island shores (N_shore (5’ end) and S_shore (3’ end)) or the island shelves (N_Shelf and S_Shelf, which are defined as 0-2 or 2-4 kilobases flanking the island, respectively. The number of probes is indicated for each island category. [file 1471-2164-15-131-S10.pptx]

## Slide 1
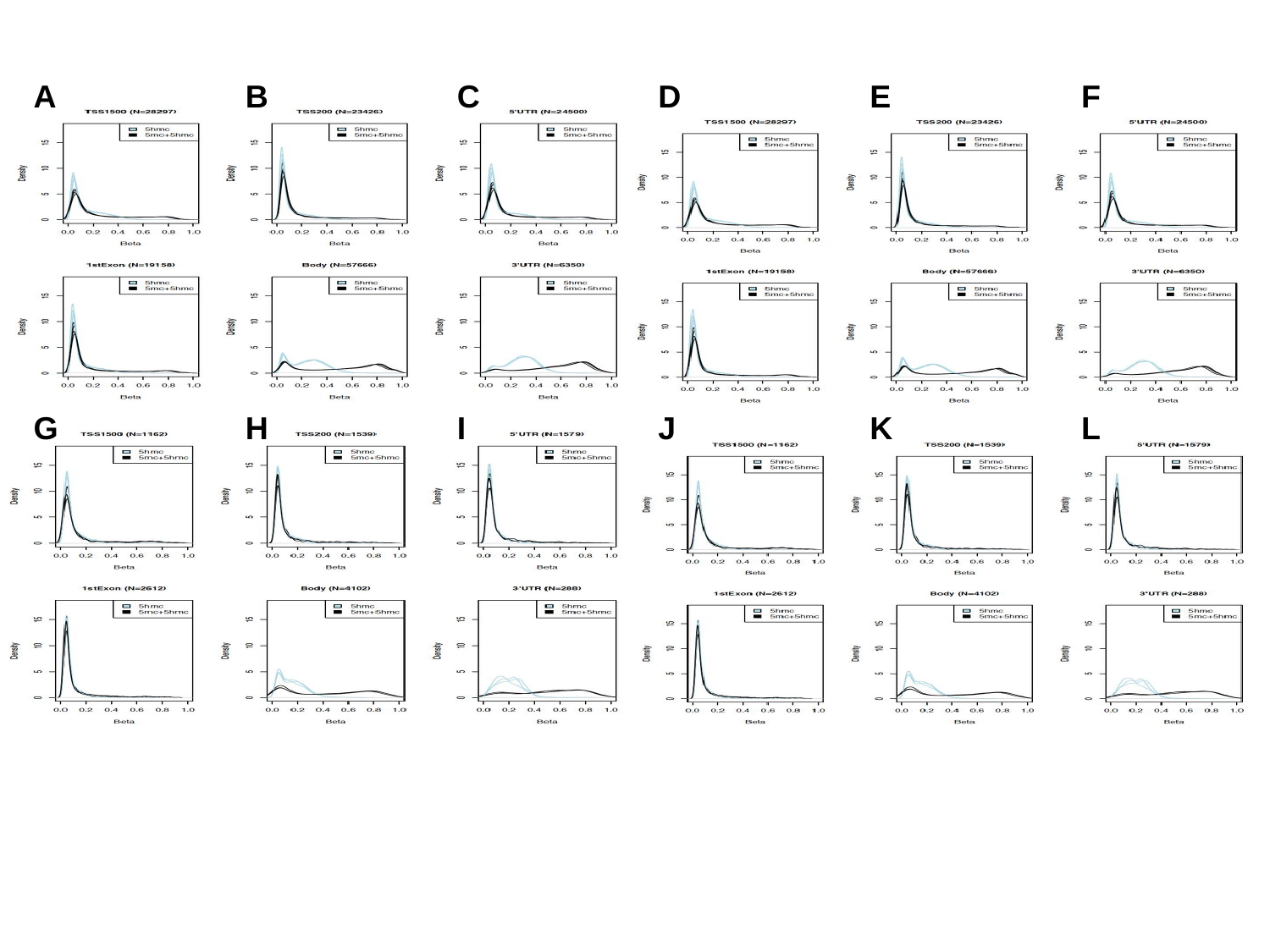

A
B
C
D
E
F
G
H
I
J
K
L

Supplement: Additional file 12 — The distribution of DNA methylation with respect to gene structures. The density (y-axis) of probes at each methylation level (x-axis; beta) in monkey (A-F) and mouse (G-L) brain samples that were either interrogated for total methylation (5mc + 5hmc (black line)) or 5-hmC levels (5hmc (blue line)). Profiles shown are delineated relative to a gene, including the distance to the gene transcription start site (TSS; TSS1500 (i.e. within 1500 bp of the TSS) and TSS200), 5’UTR, 1st exon, body, and 3’UTR. The number of probes is indicated for each island and gene category. [file 1471-2164-15-131-S12.pptx]

## Slide 1
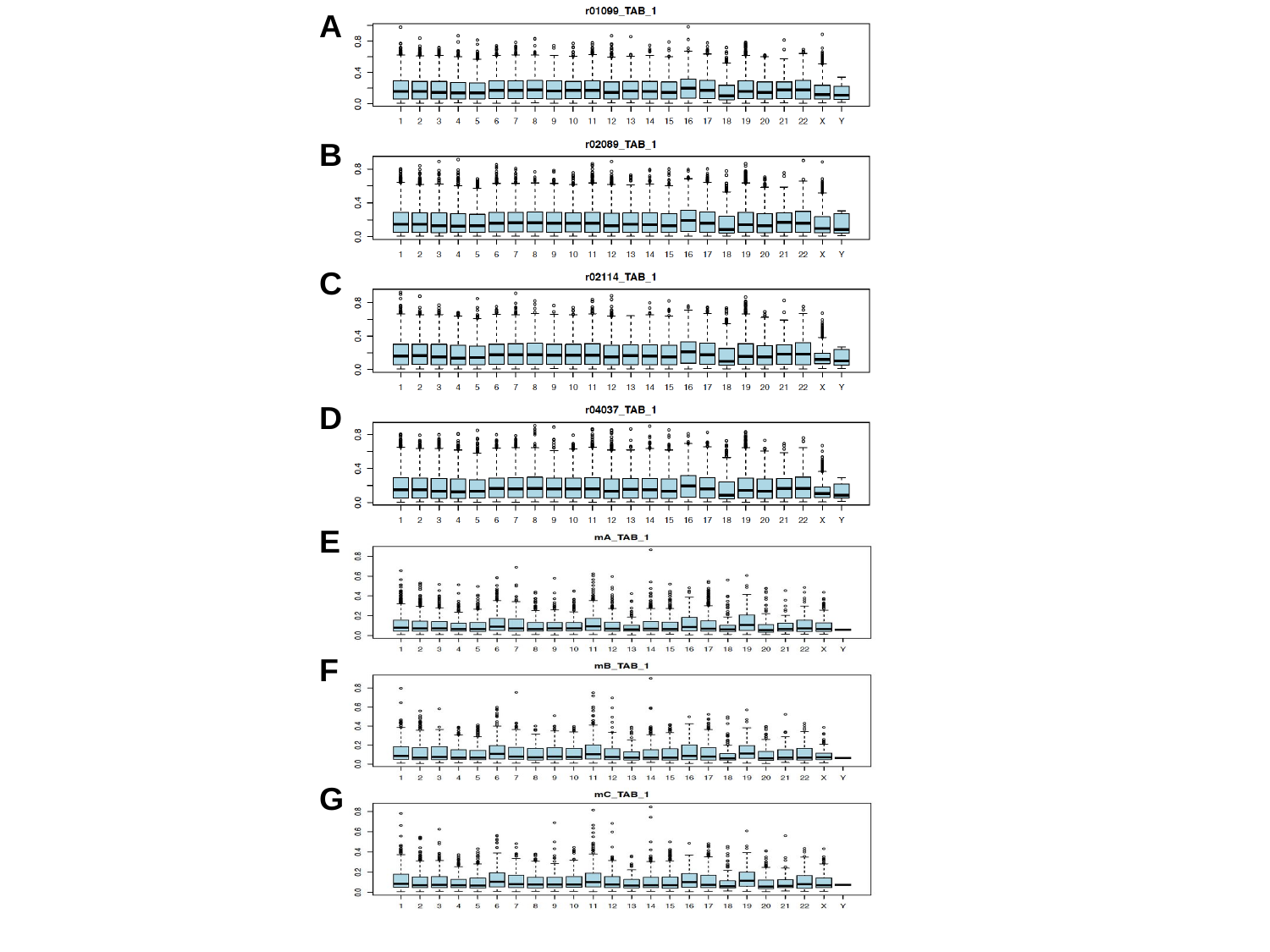

A
B
C
D
E
F
G

Supplement: Additional file 13 — The range of 5-hmC levels on each chromosome. The distribution of 5-hmC in monkey (N = 4; A-D) and mouse (N = 3; E-G) brain tissues is shown using box and whisker plots depicting the 5-hmC level (y-axis) of probes by chromosome (x-axis). [file 1471-2164-15-131-S13.pptx]
